# Understanding Comprehensive Sexuality Education: A Worldwide Narrative Review

**DOI:** 10.7759/cureus.74788

**Published:** 2024-11-29

**Authors:** Monica Albert Sekhar, Shanthi Edward, Angeline Grace, Shirley Esther Pricilla, Sushmitha G.

**Affiliations:** 1 Community Medicine, Sree Balaji Medical College and Hospital, Chennai, IND

**Keywords:** adolescent and sexual health, narrative review, public-health education, sex education, sexuality

## Abstract

Comprehensive sexuality education (CSE) is curriculum-based teaching and learning of various dimensions of sexuality. By equipping young people with accurate information on sexual and reproductive health, CSE promotes healthier populations and fosters a more informed workforce, contributing positively to national economies. Although known to have many benefits, CSE is not universally accepted or implemented. The risks of HIV/AIDS, teenage pregnancy, gender-based violence, child abuse, sexually transmitted infections (STIs), unsafe abortion, mental health challenges, gender inequality, economic burden, and persisting taboo and stigma can be minimized by providing age-accurate and culturally sensitive CSE. This review delves into the definitions and importance of sexuality and CSE, emphasizing CSE's critical need in today’s society to promote informed, healthy decisions and well-being. It explores the optimal age groups for introducing CSE, addressing age-appropriate content designed to equip young people with essential knowledge on body awareness, emotional development, relationships, and sexual health. The review further outlines the key benefits of CSE, such as reducing rates of STIs, unintended pregnancies, and gender-based violence. Finally, it provides a comprehensive overview of CSE's global status, examining diverse regional approaches, policy frameworks, and implementation challenges.

## Introduction and background

Sexuality is an essential part of human life, encompassing a broad range of expressions, behaviors, identities, and experiences. It is an intrinsic part of being human, influencing our physical and emotional health, relationships, and overall well-being. The Sexuality Information and Education Council of the United States (SIECUS), in its Board position statement, claims that human sexuality encompasses an individual’s sexual knowledge, beliefs, attitudes, values, and behaviors. It involves multiple dimensions, including the anatomy, physiology, and biochemistry of the sexual response system; identity, orientation, roles, and personality; as well as thoughts, feelings, and relationships. Sexuality is shaped by ethical, spiritual, cultural, and moral influences. In the broadest sense, all individuals are sexual [1]. The benefits of comprehensive sexuality education are well known. It provides accurate, culturally relevant, and age-appropriate knowledge about various aspects of sexuality. It helps individuals make informed decisions, thereby fostering healthy relationships, attitudes, and good health on a large scale. The clinical importance of CSE is that it leads to good long-term physical and mental health outcomes. This happens when individuals use timely healthcare services and preventive measures. Furthermore, it reduces feelings of guilt, shame, and confusion related to sexuality, which can lead to depression or anxiety if not addressed [2]. Lack of sexuality education policies leads to many harmful biological, mental, and social effects, like increased rates of sexually transmitted infections (STIs) and unintended pregnancies. Lack of conversations about sexuality tends to compound due to the social stigma attached to it making individuals vulnerable to abuse, poor relationships, and numerous misconceptions. Given the following reasons, implementing CSE is not merely an educational measure but rather a public health intervention with far-reaching benefits for both the individual and community alike [2].

## Review

Comprehensive sexuality education

Many authors talk about the multi-dimensional construct of sexuality [3,4]. The three dimensions that interact with each other to shape one’s sexuality are biological factors, psychological variables, and sociocultural variables. Understanding sexuality requires a comprehensive exploration of these various facets, which collectively shape individual and collective experiences. To understand sexuality, there is a need for the provision of the right information and awareness. CSE is a curriculum-based teaching process about various aspects of sexuality. According to SIECUS, sexual health education is something that begins at birth and must continue throughout one’s life. Access to accurate and age-appropriate sexuality education is a fundamental human right. Sexuality education should address all three dimensions within three domains: (1) cognitive learning domain (information), (2) affective learning domain (feelings, values, and attitudes), and (3) behavioral learning domain (communication, decision-making, and other skills).

Determining the optimal time to introduce sexuality health education to children is crucial. Research suggests that discussions about sexual health should begin as soon as children regularly interact with individuals outside of their immediate family (parents), that is during preschool [5]. According to the American Academy of Pediatrics (AAP) and SIECUS, initiating these conversations early from the ages of five to 10 years of age helps children develop a healthy understanding of their bodies and boundaries, and equips them with the knowledge to protect themselves from potential harm. It also lays the groundwork for future education about CSE. Not all countries have established policies or legislation to implement CSE from the preschool level, particularly as this aspect of health has only recently begun to gain significant attention. Given that SIECUS identifies achieving sexual health as a critical developmental milestone during adolescence, it becomes imperative to prioritize CSE for adolescents, the age group most directly impacted by this developmental process [6]. This underscores the urgent need to make CSE accessible to adolescents, ensuring they are equipped to achieve this milestone. The subsequent section delves into the specific reasons why young people require CSE.

Importance of CSE for adolescents

The need for investing in adolescent sexual health within a broader perspective was recognized as early as 1994 during the International Conference on Population and Development (ICPD) [7]. During this conference, it was brought to the limelight that meeting the educational and service needs of adolescents was needed to enable adolescents to deal with their sexuality responsibly and positively. However, many programs adopt a conventional risk-based approach to adolescent sexuality. This kind of approach shifts the focus only to public health indicators like teenage pregnancies, unsafe abortions, maternal mortality rates, etc. This happens despite the call for a shift of attention to “soft outcomes” like gender attitudes, relationships, and skills [8,9]. There are multiple issues that young people face that make CSE important for them, a few of which are listed next.

Puberty

The transition to adulthood is often seen as an exciting and significant change. For boys, puberty is positively linked to sexual feelings, whereas girls often encounter conflicting information about virginity, sexuality, womanhood, and fertility. Menstruation, marking the onset of puberty for girls, is often surrounded by cultural taboos and stigma. In some settings, girls are forced to be away from families and eat alone, sleep alone, or miss school while menstruating. Menstruation is generally neglected, and many girls lack proper knowledge, leading to fear, anxiety, and unpreparedness when they begin menstruating [10]. 

Pregnancy

The worldwide birth rate for females between the ages of 15 and 19 averages 49 per 1000, with national rates varying from one to 299 births per 1000 girls, according to the 2014 World Health Statistics. Early marriage is a crucial element for these birth rates [11]. In developing nations, marriage accounts for over 90% of the births to teenage moms [12]. Early pregnancy and childbirth are the second leading cause of death for girls under the age of 19, and they can have detrimental effects on one's health and social standing. In India, according to the National Family Health Survey (NFHS) 5 data, it was seen that nearly 8% of adolescent girls are already mothers between the ages of 15 and 19 [13]. Teenage girls who are pregnant are more likely to stop their studies and drop out of school, which will limit their options for employment and other aspects of life in the future [14].

Access to Modern Contraception

Although unmet needs for contraception among married women are known in most countries, the unmet need among unmarried adolescents is often not brought to light owing to the hesitation of adolescents, especially in traditionalist societies, to confess that they are sexually active [15]. In Africa and Asia, significant information gaps exist regarding where to obtain and use various traditional contraceptive methods, such as condoms and emergency contraception, as well as where to access pregnancy or HIV testing facilities [16].

‌Unsafe Abortions

Reports from WHO show that every year, 3 million girls undergo unsafe abortions at the ages of 15 to 19 years of age [17]. This problem is aggravated, especially due to restrictions to accessing safe abortion in many parts of the world. Due to this reason, many adolescents resort to unsafe abortions performed by unskilled providers or quacks. This causes a significant number of deaths in this population [18].

Gender-Based Violence

One out of every three women worldwide has experienced physical or sexual violence, either from a partner or someone else, during their lifetime. Facing violence increases the risk of unintended pregnancies, abortion, HIV infection, and other social problems. Child sexual abuse affects both boys and girls [19]. A review showed that 8% to 31% of girls and 3% to 17% of boys faced sex abuse as a child [19].

HIV/AIDS

In sub-Saharan Africa, only 29.8% of young women and 36.4% of young men have basic HIV protection knowledge. Among youth between the ages of 15 and 24 in Western and Central Africa, it was found that less than 31% of young men and 23% of young women have comprehensive and accurate knowledge about HIV prevention and condom use [20]. A study showed that fewer than one-third of participants reported having correct knowledge of HIV/AIDS; this disparity in knowledge was associated with rural residence, poverty, and low levels of education [21].

STIs

The World Health Organization (WHO) states that each year there are about 333 million new cases of curable STIs being detected. The highest rates of these infections happen among those aged 20-24 years, followed closely by those between 15 and 19 years. One out of every 20 people contract an STI other than HIV/AIDS each year [22].

Institutions providing CSE

Many institutions act as platforms for providing sexuality health education to adolescents, including families, schools and educational institutions, healthcare providers, government programs, nonprofit organizations, community centers, the internet, and social media. Among these, the education sector is of prime importance for providing CSE [2]. With existing infrastructure and skilled teachers who are trusted sources of information for students, schools offer a great opportunity to implement a CSE curriculum within an existing framework. A school setting is ideal for dispensing CSE for several reasons. Since most children aged five to 13 spend significant time in school, schools serve as a viable means to reach a large and diverse group of children and adolescents effectively, in replicable and sustainable ways. Also, most young people experience puberty and adolescence while in school, going through their first relationships and possibly their sexual debut during this time. Schools already have the structure or framework to provide age-appropriate CSE topics that build on previous content, making them a convenient source for CSE delivery. Given the sensitive nature of these topics and the necessity to provide a safe environment for adolescents, school authorities can regulate settings to ensure they are protective and supportive. Children are likely to feel more at ease within familiar surroundings than if they were required to attend classes in a new location. Furthermore, schools serve as a link between children, families, and parents, acting as major support system centers that can monitor the physical, mental, and sexual health of adolescents [2].

Content of CSE

The International Planned Parenthood Federation (IPPF) and the Asian-Pacific Resource and Research Centre for Women (ARROW) identify seven essential components of CSE: gender, sexual rights and sexual citizenship, sexual and reproductive health (SRH) and HIV, pleasure, violence, relationships, and diversity [23,24]. According to IPPF, the development of educational materials for CSE must adhere to principles of good practice, which are categorized under four main headings: planning, delivery, assessment, and evaluation. Within the assessment and evaluation category, conducting pre- and post-test assessments is emphasized as a crucial practice. These assessments provide an opportunity to identify the needs of individuals and offer a platform for learners to reflect on what they have learned, thereby enhancing their understanding of the topic.

The United Nations Educational, Scientific and Cultural Organization (UNESCO) in collaboration with other agencies like UNICEF, the joint United Nations Population Fund (UNFPA), and WHO has developed the International technical guidance on sexuality education (ITGSE) [2]. This guidance supports education, health, and other relevant authorities in designing and implementing CSE programs and materials for both in-school and out-of-school settings. CSE as per the ITGSE guidelines should be scientifically accurate, gradually introduced according to age and development, based on a curriculum, thorough and all-encompassing, grounded in human rights, focused on gender equality, culturally relevant and suited to the context, transformative and designed to develop life skills for making healthy choices [25]. ITGSE divides the CSE curriculum into eight key concepts, which are organized into knowledge, attitudinal, and skill-based objectives. These objectives are divided into four age groups: 5-8 years, 9-12 years, 12-15 years, and 15 to 18+ years, for primary and secondary school students. The learning objectives are designed to be age-appropriate, with simpler concepts for younger students and more complex ones for older students [2].

Benefits of CSE

CSE offers numerous benefits, a few of which are listed in Table 1.

**Table 1:** Benefits of providing comprehensive sexuality education.

Benefit	Description
Enhances knowledge and attitudes	Improves understanding of sexuality, enabling informed and mature decisions regarding sexual and reproductive health [2]
Reduces risk-taking behaviors	Lowers the likelihood of engaging in risky sexual behaviors [26]
Delays sexual debut	Encourages adolescents to wait longer before engaging in sexual activity [26]
Decreases rates of STIs, including HIV	Reduces the prevalence of sexually transmitted infections and HIV [26]
Lowers unintended pregnancies	Programs focusing on abstinence and gender issues help prevent unintended pregnancies [27,28]
Reduces child abuse	Helps prevent and address instances of child abuse by providing critical knowledge [29-33]
Corrects misinformation	Counters myths and incorrect information often spread by unreliable sources due to stigma [29-33]
Promotes condom and contraceptive use	Increases awareness and proper use of condoms and contraceptives [2,26-27]
Fosters respect for women and gender sensitivity	Encourages a respectful attitude toward women and promotes awareness of gender equality and body autonomy [2,26-27]
Promotes social justice and inclusivity	Improves understanding of gender norms, equity, rights, and social justice, contributing to a more inclusive society [2,34-35]

CSE across different areas

Western World

The introduction of CSE in schools dates back 50 years, with Western Europe leading the cause [2]. Sex education across the Western world exhibits significant variation by country, state, and even locality, reflecting diverse cultural, political, and religious influences. Despite these differences, several common themes emerge across the region [25]. Certain countries like Sweden, Norway, and the Netherlands have an existing curriculum/sexuality health education program in schools [2]. 

In the United States, sex education is not standardized, leading to significant variation within each state. Certain states mandate comprehensive programs, while others emphasize abstinence-only education, which promotes abstinence until marriage and often excludes information about contraception and safe sex practices [36]. In Canada, sex education generally follows a comprehensive approach mandatory at all publicly funded schools. Challenges faced by adolescents in this country include accessing health care services i.e., where to get contraceptives, go for testing STIs, etc. Western European countries generally adopt a comprehensive and progressive approach to sex education, starting at a young age and covering topics like consent, gender identity, sexual orientation, and relationships. This early and inclusive education is often credited with the low rates of teenage pregnancies and STIs seen in countries like the Netherlands and Sweden [37].

Asia and Pacific Regions

A quarter of the population in the Asia and Pacific region is made up of adolescents. While adolescent biological needs are generally consistent across populations, they are influenced by diverse sociocultural, political, and economic contexts as adolescents transition into adulthood in different regions of the world. The Asia and Pacific regions as a whole are characterized by shared challenges, such as conservative sociocultural norms and societal attitudes that restrict unmarried individuals' access to SRH information and services. Despite these challenges, it is to be noted that studies show that 22% of unmarried girls and 41% of unmarried boys have engaged in sexual activity [38]. Although national data are not available in this context, other studies show that at least three-quarters of 15- to 24-year-olds have had sex before marriage [39].

Bangladesh’s constitution promotes equality and non-discrimination, with the 2012 population policy emphasizing family planning and reproductive health awareness. The 2006 National Adolescent Reproductive Health Strategy supports married adolescent girls but lacks support for unmarried youth [40,41]. No studies on unmarried girls' sexual behavior exist, but 13% of unmarried male adolescents reported sexual activity, linked to peer pressure [39,42]. Unmarried adolescents face a 35-fold higher abortion incidence [43]. A rural survey of 5,106 girls aged 13-19 years found that 43% experienced sexual harassment [44]. Among girls aged 10-19 years, only 20% were aware of STIs, 60% were unaware of HIV/AIDS, and 48% did not know the routes of HIV transmission [45].

In Mongolia, the National Program of Action for the Development and Protection of Children emphasizes improving adolescents' awareness of education, reproductive health, HIV/AIDS prevention, and space births. There is no component of CSE in their laws and policies [46]. Since 2018-2019, reproductive and sexuality health education has become a mandatory subject. However, the SRH curriculum is not comprehensive, and teacher training is insufficient. Despite strides in sexual health education, insufficient coordination and funding hinder comprehensive service delivery [47]. Low awareness of reproductive and sexual health persists, with less than 40% of adolescents having accurate family planning information and under 20% having comprehensive STI/AIDS prevention knowledge. The age of sexual debut is decreasing [48].

Various policies in Nepal mention components of CSE, though not comprehensively [49]. The Second Long-Term Health Plan (2017) emphasizes the provision of adolescent reproductive health services. The Environment, Health, and Population (EPH) curriculum, mandatory for students, includes CSE and reproductive health education but becomes optional in classes 9 and 10 [50]. This curriculum only addresses in-school youth, leaving out-of-school adolescents without provision. The CSE is not comprehensive, lacking coverage on gender, human rights, violence, sexual rights, pleasure, and diversity, and is often taught within a heteronormative framework [51].

China's laws and policies reference limited components of CSE, focusing on puberty, sexual health, and STIs, with an emphasis on abstinence. Implementation varies among institutions, and the country’s latest strategic education document excludes CSE [39,52]. Studies reveal conservative attitudes, poor SRH awareness, and low knowledge levels among females, with many preferring online education [53]. Adolescents, especially those living with HIV, rarely discuss sexuality with their parents, and many lack awareness about condoms and HIV [54]. Additionally, 14% of youth have experienced sexual violence, and 43% were diagnosed with STIs [55].

In Pakistan, there is no formal CSE curriculum, and provincial implementation strategies lag post-18th Amendment devolution. Youth policies in Punjab and Khyber Pakhtunkhwa have incomplete implementation plans. Civil society has integrated some CSE components into life-skills modules in Sindh and Punjab, but challenges persist. Punjab has adolescent-friendly health centers, and Khyber Pakhtunkhwa's *Jawan Markaz* offers youth counseling, though CSE coverage is unclear. HIV/AIDS prevention strategies exist but face execution difficulties due to the hostile socio-political climate [39]. There is limited data on premarital sex among adolescents, and few studies on SRH. A rural study showed that males were more knowledgeable than females about puberty, pregnancy, family planning, and STIs [56]. Adolescents in Pakistan face limited access to SRH services. This issue is reflected in problems like early marriages, gender discrimination, violence, unintended pregnancies, low contraception rates, etc. The Islamic context and societal taboos surrounding sexuality hinder open discussions here and further perpetuate misconceptions [57]. 

Sri Lanka has various programs supporting education, gender equality, health, HIV/AIDS, and youth issues. The Ministry of Health and the National Institute of Education launched the Reproductive Health Education Program to enhance SRH knowledge. Schools include an adolescent SRH component, and the National Youth Services Council offers counselling for out-of-school youth. The existence of a Policy Framework and National Plan of Action to address sexual and gender-based violence underscores the importance of awareness and education. A study revealed poor SRH knowledge among adolescents, with health professionals preferred for information, and only 34% of adolescents discussing sexual issues with their parents [58].

SRH is a critical component of public health, especially in a country like India where cultural taboos and stigmas often hinder open discussions on the topic. Although the government of India has taken many initiatives to address the reproductive health of women, implementation of comprehensive sexual health education measures for unmarried adolescents or teens is still questionable. Also, sex education in schools had strong objections from even parents, teachers, and politicians, leading to its ban in six states: Maharashtra, Gujarat, Rajasthan, Madhya Pradesh, Chhattisgarh, and Karnataka [59]. While India has implemented various reproductive health programs, there is currently no specific policy or legislation that supports CSE at a national level. This lack of formal policy has created challenges in accepting and institutionalizing new, informal sources of CSE across many states which is evident in the ban across certain states towards sex education. As a result, there is inconsistency in how sexual health education is delivered, often leading to regional disparities in access to accurate and comprehensive information on SRH. Without a national framework or clear guidelines, local governments and educational institutions may struggle to incorporate CSE into their curricula, which can hinder efforts to address adolescent sexual health needs effectively [60].

Recommendations

Commitment to CSE Policy and Legislation

Governments must prioritize the integration of CSE into national policies and legislation. The absence of clear, enforceable policies hinders the widespread implementation of CSE. Political will is crucial, and leaders must recognize the importance of sexual health education in achieving long-term social and public health goals. Countries should adopt comprehensive policies that ensure CSE is an essential part of the educational framework. This will require dedicated efforts to overcome resistance, address misconceptions, and promote the benefits of CSE.

Early Implementation

CSE should be introduced at the earliest possible stage in education, ideally beginning in preschool, where children can learn about personal boundaries, respect, and body autonomy. As children mature, the content should evolve in complexity, covering topics like relationships, consent, gender identity, sexual health, and rights in a developmentally appropriate manner. This early start ensures that children are well-equipped with the knowledge they need to make informed decisions as they grow. Culturally relevant content must be incorporated to respect local values and norms, while still providing accurate and comprehensive information.

Integrated Approach

For CSE to be effective, it must be delivered in an integrated manner, where schools, families, and communities collaborate. Educators should not work in isolation; rather, family engagement and community involvement are essential to reinforcing lessons learned in school. Such collaboration can help create a supportive environment for adolescents and reduce stigmas surrounding sexual health. Involving parents and guardians in the educational process ensures that key messages are reinforced at home, which is critical for the success of CSE at the grassroots level.

## Conclusions

This review underscores the need for CSE, especially given its critical role in preventive healthcare and overall well-being. In the Western world, Europe generally promotes CSE from a young age, which is linked to low rates of teenage pregnancies and STIs. The United States and Canada show variability, with Canada favoring a comprehensive approach. In Asia, CSE faces resistance due to conservative norms, limiting access to SRH information. Countries like Bangladesh, Mongolia, and Nepal have partial policies, while China and Pakistan emphasize abstinence and face sociopolitical barriers. In India, strong cultural taboos impede CSE, with bans in multiple states and limited adolescent-focused sexual and reproductive health education measures, especially for unmarried teens. The country also suffers from a lack of policy and framework for CSE. It can be concluded that given the need for CSE in today’s time, significant efforts are still required in many countries where political will and societal support remain barriers to CSE adoption, especially in the Asian continent. CSE should be integrated as an essential part of education, ensuring that young individuals everywhere have access to knowledge that supports responsible decision-making, health protection, and lifelong well-being.
